# Effect of aging on marginal and internal adaptation of bulk-fill resin composites with different viscosities in class II restorations

**DOI:** 10.1186/s12903-026-10102-1

**Published:** 2026-11-05

**Authors:** Asmaa Saad Elsaeed, Youniss Saleh Harp, Hamdi Hamama, Salah Hasab Mahmoud

**Affiliations:** 1 Conservative Dentistry Department, Faculty of Dentistry, Mansoura University, Mansoura, 35516 Egypt; 2 Department of Basic Dental Sciences, Faculty of Dentistry, Ibn Sina University for Medical Sciences, Amman, 16197 Jordan; 3 Faculty of Oral and Dental Medicine, Delta University of Science & Technology, Gamasa, Egypt

**Keywords:** Bulk-fill composite, Pre-heating, Sonicfill composite, Thermoviscous composite, Interface analysis, Marginal quality

## Abstract

**Aim:**

To evaluate and compare the effect of thermocycling and water storage on the marginal and internal adaptation of bulk-fill resin composites with different viscosities (No-cap low viscosity, thermoviscous, sonically activated, and conventional bulk-fill) in Class II restorations using scanning electron microscope.

**Materials and methods:**

Fifty-six sound molars were prepared with standardized class II cavities (occlusal depth:3.0 mm, bucco-lingual width: 3.0 mm, depth, proximal box: 3.0 mm bucco-lingual width, 1.5 mm axial depth, and gingival margin extended 1-mm below CEJ). They were assigned into four groups according to the material used (*n* = 14). For the marginal and internal adaptation test, half of the specimens in each group were tested after one week, however the remaining half were tested after 12 months of water storage and exposure to 10,000 thermal cycles between 5 and 55 °C. Replicas were made and evaluated for marginal and internal adaptation using a scanning electron microscope at different magnifications. Continuous margin percentage values (%CM) and Maximum gap width values (MGW) were statistically analyzed.

**Results:**

Aging did not significantly affect external % CM, internal % CM, or external MGW (*p* > 0.05). However, internal MGW values were significantly lower in immediate specimens compared with aged ones (*p* < 0.05). Among the tested composites, no significant differences were observed in external or internal % CM or external MGW (*p* > 0.05). Nevertheless, at the dentin gingival margin, lower-viscosity bulk-fill composites generally showed numerically lower internal MGW values than the high-viscosity bulk-fill composite. However, after Bonferroni adjustment, statistically significant differences were observed only between Flowable and Tetric and between SonicFill and Tetric (*p* < 0.05). Furthermore, a strong correlation was detected between external and internal adaptation parameters (%CM and MGW) (*p* < 0.05).

**Conclusion:**

Aging did not adverse effect %CM or external MGW but significantly increased internal MGW. Some lower-viscosity bulk-fill composites showed lower internal MGW values at the gingival margin than the high-viscosity composite under the conditions of this in vitro study.

**Supplementary Information:**

The online version contains supplementary material available at https://doi.org/10.1186/s12903-026-10102-1.

## Introduction

A key determinant of the long-term success of any restorative material is its ability to achieve optimal adaptation to external or internal cavity surfaces [1]. Resin composite is considered the material of choice for direct restorations. A major drawback is polymerization shrinkage and the associated stresses, which hinder adequate adaptation and contribute to defects at the marginal and internal interfaces of restorations. These stresses can cause postoperative sensitivity, marginal discoloration, and secondary caries [2]. 

Many strategies have been proposed to mitigate the effects of polymerization shrinkage, including the use of various light-curing protocols [3], the use of a resin–ionomer base in a sandwich technique [4] and incremental layering approach [3, 5]. Incremental layering approach improves light penetration and ensures proper polymerization, thereby reducing polymerization stresses and improving adaptation to cavity walls [5, 6]. However, this technique has some limitations, including the potential for air trapping between resin composite layers and time-consuming [7, 8]. 

Bulk‑fill resin composites were introduced to overcome the limitations of the incremental layering approach [9]. These materials can be placed into cavities in a single increment up to 4–6 mm. They are available in different viscosities, high-viscosity (body bulk-fill) and low-viscosity (flowable bulk-fill) types [10]. High-viscosity types have better mechanical properties but adapt poorly, while low-viscosity types adapt better but have inferior mechanical properties, requiring capping with a high-viscosity resin composite [11–14]. 

Manufacturers have developed a sonic-activated bulk-fill resin composite to improve adaptation, where sonic energy reduces viscosity during placement, improving adaptation [15–18]. Similarly, preheating high-viscosity resin composite increases flow and decreases marginal gaps as reported in previous studies [12, 19–21]. Recently, VisCalor bulk was introduced as a thermoviscous composite; its viscosity decreases by more than 90% at 69 °C, providing the combined advantages of flowable and packable composites [22]. 

Several studies [14–28] have evaluated the marginal adaptation of bulk-fill resin composites under different aging conditions, primarily using thermocycling and water storage. However, previous investigations have generally focused on individual materials or limited comparisons between selected composites, and the direct comparative behavior of different viscosity and handling strategies under identical experimental conditions remains insufficiently characterized. To the best of our knowledge, no previous study has directly compared no-cap low-viscosity, thermoviscous, sonic-activated, and conventional high-viscosity bulk-fill resin composites within the same experimental framework while simultaneously evaluating both external and internal adaptation before and after artificial aging. This comparison is relevant because differences in viscosity and handling characteristics may influence not only external marginal integrity but also internal adaptation, and these two aspects may not respond similarly to aging. Accordingly, this study aimed to evaluate and compare the effects of thermocycling and water storage on the external and internal adaptation of four bulk-fill restorative systems representing different viscosities (no-cap low-viscosity, thermoviscous, sonic-activated, and conventional high-viscosity) in Class II restorations. Additionally, replica-based SEM analysis was used to assess the percentage of continuous margins (% CM) and maximum gap width (MGW) at external and internal interfaces, enabling a comprehensive comparison of adaptation and its response to artificial aging within the same experimental framework. The research null hypotheses were as follows: (1) Thermocycling and water storage would not significantly affect external marginal or internal adaptation of bulk-fill resin composites, as measured by percentage of continuous margin (%CM) and maximum gap width (MGW). (2) The tested bulk-fill resin composites and the type of tooth substrate would not significantly affect external marginal adaptation (%CM and MGW) (3) The tested bulk-fill resin composites would not significantly affect internal adaptation (%CM and MGW). (4) There would be no significant relationship between external marginal and internal adaptation measurements (%CM and MGW).

## Materials and methods

### Materials

Four bulk-fill resin composite restorative systems with different viscosities were used in the current study, each with its corresponding manufacturer-recommended adhesive system, according to the manufacturers’ instructions. The bulk-fill resin composite systems were; No-cap low-viscosity bulk-fill resin composite (PALFIQUE BULK FLOW / PALFIQUE BOND, Tokuyama, Tokyo, Japan), thermoviscous bulk-fill resin composite (VisCalor bulk / Futurabond U, VOCO; Cuxhaven, Germany), sonic-activated bulk-fill resin composite (SonicFill3/ OptiBond Universal, Kerr, Orange, CA, USA), and high-viscosity bulk-fill composite ( Tetric N-Ceram Bulk Fill /Tetric N-Bond Universal Adhesive (Ivoclar, Vivadent, Schaan, Liechtenstein), The full description of all materials is summarized in Table 1.

**Table 1:** Materials used in the study

Material	Specification	Composition	Manufacturer’s Instructions	Manufacture	(lot number)	Shade
PALFIQUE^®^ BULK FLOW	No-cap Nanohybrid flowable bulk-fill Composite	Resin matrix includes: Bis-GMA, TEGDMA, Bis-MPEPP, Mequinol (a stabilizer /UV absorber), Dibutyl hydroxyl toluene (BHT; antioxidant) UV absorber (unnamed specific compounds) Filler: Spherical silica-zirconia (SiO₂-ZrO₂) filler, Filler load: ~70% by weight.	Dispense Up to 4 mm directly into cavity using compule; adapt with hand instrument; no capping layer required; finish and polish after curing. 20 s (≥ 1000 mW/cm²)	Tokuyama, Tokyo, Japan	125EY1	A2
PALFIQUE BOND	self-etch bond	6-methacryloyloxyhexyl 2-thiouracil- 5-carboxylate (MTU-6). γ-methacryloxypropyl triethoxy silane (γ-MPTES). HEMA (2-Hydroxyethyl methacrylate), Bis-GMA, TEGDMA. Solvent (Acetone).	1. Apply the adhesive to the entire cavity surface using a micro brush and scrub for 10 s. 2. Gently air-dry for 5 s to evaporate solvent until the adhesive film appears thin and glossy. 3. Light cure for 10 s.	Tokuyama, Tokyo, Japan	175EY2	
VisCalor Bulk	Thermo-viscous Nanohybrid bulk-fill Composite	Resin matrix includes; Bis-GMA, UDMA, TEGDMA Filler; Glass ceramic filler, Silicon dioxide nanoparticles (20–40 nm) Filler load: ~83% by weight.	Preheat material to 68 °C using VisCalor dispenser; inject Up to 4 mm into cavity; adapt and sculpt after cooling; single increment placement. 20 s (≥ 1000 mW/cm²)	Vocco, Cuxhaven, Germeny	22,220,693	A2
Futurabond U	Universal adhesive	HEMA and BIS-GMA, UDMA, Acidic adhesive monomer (as 10-MDP or similar phosphate monomer), HEDMA as a diluent monomer. Solvent: Ethanol. Silica nanoparticles (as filler)	1. Activate the single-dose capsule and mix for 10 s. 2. Apply adhesive to the prepared surface and rub for 20 s. 3. Gently air-dry for 5 s until a uniform, glossy film is obtained. 4. Light cure for 10 s.	Vocco, Cuxhaven, Germeny	18,077,614	
SonicFill3	Sonic-activated bulk-fill Nanohybrid bulk-fill Composite	Matrix; EBPDMA, Bis-GMA, Bis-EMA and TEGDMA Filler; zirconium oxide and silica oxide Filler Load: 83.5% by weight.	Apply using SonicFill handpiece with sonic activation; flow Up to 5 mm into cavity; stop activation for sculpting; finish after curing. 20 s (≥ 1000 mW/cm²)	Kerr Corp, Orange, CA, USA	9,049,510	A2
OptiBond Universal	Universal Adhesive	HEMA, Glycerol dimethacrylate, GPDM adhesive monomer technology. Solvent: Acetone	1. Apply adhesive with a rubbing motion for 20 s. 2. Gently air-dry for 5 s to evaporate solvent until the surface is thin and glossy. 3. Light cure for 10 s.	(Kerr Corp, Orange, CA, USA)	10,550,195	
Tetric N-Ceram Bulk Fill	Nanohybrid high-viscosity bulk-fill composite	Matrix; Bis-GMA, Bis-EMA, UDMA, Filler; glass oxide, ytterbium trifluoride barium glass., Filler loading: 75–77% by weight.	Apply Up to 4 mm in increments; adapt with hand instrument; use Tetric N-Bond Universal adhesive prior to placement. 10 s (≥ 1000 mW/cm²)	(Ivoclar, Vivadent, Schaan, Liechtenstein)	Z02SV4	IVB

## Methods

### Sample size calculation

Sample size was calculated based on previous research (Agarwal et al., 2024) [18]. Using G*Power program version 3.1.9.7 (Faul et al., 2007) [29] to calculate sample size based on effect size of 0.722, using 2-tailed test, error (α) = 0.05 and power (1-β) = 95%, the calculated sample size was 7 in each subgroup.

### Ethical considerations and teeth selection

Caries-free, intact, freshly extracted human maxillary molars were selected in this current study. Teeth were extracted due to periodontal disease from patients ranging in age from 45 to 55 after getting their approval through written informed consents. The protocol of the current study was approved by the Ethics Committee of Faculty of Dentistry, Mansoura University under number A02011023 CD.

All teeth were examined macroscopically and microscopically at 20× magnification (Labomed Megna, Model 6129001, Labo-America, Inc., Fremont, CA, USA) to exclude cracks or defects. Teeth were cleaned of any soft tissues or hard deposits using a hand scaler (Nordent, Ivory #2–3, USA). The teeth were then washed under running water and disinfected in a 0.5% Chloramine-T solution for 48 h. Teeth were polished using oil- and fluoride-free (whip mix, Louisville, USA) and rubber Cups (Shofu, One Gloss, Japan) rotated in a low-speed handpiece. Finally, teeth were stored in distilled water at 37 °C in an incubator (BTC, Cairo, Egypt).

### Study design

In the current study, 56 extracted human maxillary molars were selected. The teeth were assigned to four different experimental groups (*n* = 14) according to the restorative system used. Then, each material group (*n* = 14) was randomly divided into 2 subgroups, (*n* = 7) according to the aging conditions [30] either (1) kept in distilled water for 1 week at 37 °C, or (2) stored in sterile water for 12 months at 37 °C in an incubator with the water changed weekly, then thermocycled for a total number of 10,000 cycles, which approximately represents 12 months of clinical service before testing.

### Teeth mounting and preparation

Molars were vertically fixed in acrylic resin blocks up to 3 mm below cemento-enamel junctions (CEJ ) (Acrostone, Egypt) to simplify handling during restoration procedures. All cavity preparations were performed by a trained operator. With the help of a digital cavity preparation machine, standardized occluso-mesial Class II cavities were prepared in all molars using cylindrical medium grit diamond bur (K881 012, öko DENT, Germany) and finished with a 25 μm finishing diamond under copious water coolant with a high-speed handpiece (W&H, RC-90RM, Austria).

The occlusal cavity dimensions were 3 mm in bucco-lingual width and 3.0 mm depth, while the proximal box had 3.0 mm in bucco-lingual width, 1.5 mm in axial depth, and the gingival margin extended 1 mm below CEJ [31]. All cavity margins were not beveled, with slightly rounded line angles. The bur was changed after every five cavity preparations. A single operator carried out all cavity preparation and restoration procedure using 4× magnification and LED headlight illumination (Amtech, Wenzhou, China).

### Restorative procedures

After cavity preparation, cavities were cleaned and dried. Occlusal and proximal enamel margins of the cavities in all groups were selectively etched with 37% phosphoric acid (N-Etch, Ivoclar Vivadent, Amherst, NY, USA) for 15 s, rinsed with water for the same duration, and gently dried with oil-free air without desiccation. Adhesive systems were applied according to the assigned group, following the manufacturer’s instructions shown in Table 1. Then, the air was used to thin the adhesive layer, and the adhesive was light-cured (Elipar Deep Cure, 3 M ESPE, St. Paul, MN, USA) operating at 1,000 mW/cm2 as recommended by the manufacturer’s instructions. A radiometer (Bluephase Meter II, Ivoclar Vivadent) was used to check LED curing light intensity.

A Tofflemire matrix band (Fintrec dead soft matrix, Pulpdent Corp., MA, USA) was adapted and fitted around each tooth, ensuring that the band’s end was beyond the gingival margin of the cavity to avoid the formation of gross marginal discrepancies. Then, each group was restored using the group-specific restorative material in a bulk technique as mentioned in Table 1. After removing the matrix band, the proximal surface was light-cured for 20 s.

Before the finishing and polishing steps, all specimens were kept in distilled water at 37 °C for 24 h in an incubator. Aluminum oxide discs (Extra-Thin Sof-Lex discs, 3 M ESPE) were used for restoration finishing and polishing, and for removing any visible overhangs using a low-speed handpiece (Strong 204, Daegu, South Korea) under water cooling. All specimens were removed from their fixation blocks, ultrasonically cleaned, and an epoxy resin replica was prepared before testing.

### External marginal adaptation evaluation

The mesial restored surfaces of all teeth were cleaned with ethanol and dried. Then, these surfaces were recorded using fast-setting addition silicone impression materials in heavy-body and extra-light viscosities (Aquasil Ultra+, Dentsply Sirona, USA) through a two-stage double-mix impression protocol using a two-step technique. After setting, the impressions were gently detached from the tooth specimens in line with the manufacturer’s guidelines, and subsequently stored for 12 h to ensure complete polymerization. The molds were then filled with extra-fast-curing epoxy resin (Hardman Double/Bubble, Ellsworth Adhesives, USA), while a vortex mixer (VWR, USA) was applied to minimize air entrapment and void formation. The resin casts were left to cure under ambient conditions for 24 h. Once fully set, the epoxy replicas were separated from the silicone impressions, trimmed to remove excess material, and cleaned with ethanol. The replicas were finally examined for surface integrity and the absence of defects before microscopic evaluation.

Replicas were mounted on aluminum stubs and sputter-coated with gold (Luxor Benchtop, Nanoscience Instruments, AZ, USA). They were examined under a scanning electron microscope (SEM) (JSM-6510LV, JEOL Ltd., Tokyo, Japan) at 25–500×magnifications to evaluate external marginal adaptation at proximal enamel and dentin margins. All SEM examinations and measurements were performed by a single experienced operator, blinded to the restorative procedures. Intra-examiner repeatability was assessed by repeating the assessment and remeasuring the relevant marginal adaptation parameters (%CM and MGW) under the same conditions. Marginal integrity was represented as the percentage of continuous margin (%CM) and maximum gap width (MGW) (µm). The margin was initially examined at 25× magnification. Each of the restorative material/ proximal enamel dentin interfaces was examined at 200×. Finally, gaps were analyzed and computed at 500×. Images were analyzed via image analysis software (SEM Control User Interface Ver 3.10, JEOL Ltd.). To avoid pseudoreplication and ensure statistical independence, multiple measurements obtained from each specimen were averaged to generate a single representative value per tooth. Each tooth was therefore considered the experimental unit for statistical analysis.

### Internal marginal adaptation evaluation

All teeth were fixed vertically in acrylic blocks from their distal side. Each tooth was sectioned longitudinally in the mesiodistal direction using a slow-speed diamond saw (Isomet 4000, Buehler, Lake Bluff, IL, USA) under water cooling. One cut was made per tooth, yielding two slices. Epoxy resin replicas were prepared for each slice. The adaptation at the restorative material/gingival and axial interfaces was assessed using SEM under the same parameters as external marginal adaptation. For each specimen, two internal surfaces were examined, and their values were averaged to obtain a single representative value per specimen for statistical analysis.

### Statistical analysis

The data were statistically analyzed using SPSS (SPSS version 20, IBM, Chicago, IL, USA). The % CM values for both external and internal adaptation were proved to be normally distributed after they were subjected to the Shapiro–Wilk test. However, MGW values externally and internally were not normally distributed. A two-way analysis of variance (ANOVA) was used to assess the effects of restorative materials, aging, and their interaction on %CM for both external and internal adaptation. In the case of a significant ANOVA result, post-hoc pairwise comparisons were performed using Tukey’s HSD test. The Kruskal–Wallis test was used to determine the effect of different viscosities of restorative material and aging on both external and internal MGW, in case of a significant effect, Mann–Whitney U test was used. Finally, Spearman correlation coefficients (r) tests were used to correlate %CM and MGW values in external and internal marginal adaptation tests, and to correlate between external and internal adaptation values.

## Results

### Test of normality

Shapiro–Wilk test results revealed that %CM values for both external and internal marginal adaptation were normally distributed (*p* > 0.05), whereas MGW values were not normally distributed (*p* < 0.05).

### External marginal adaptation test results

The two-way ANOVA revealed no statistically significant effects of restorative material, aging, or their interaction on the percentage of continuous margins (% CM) for either enamel or dentin margins (*p* > 0.05) Table 2. Furthermore, comparison between the two substrate types (enamel vs. dentin) using a t-test revealed no statistically significant difference in external % CM (*p* = 0.32).

**Table 2:** Results of Two-way ANOVA test of external percentage of continuous margin across materials and aging

Substrate	Source	Type III Sum of Squares	Df	Mean Square	F	Sig.	Partial Eta Squared η²
Enamel	Restoration	65.386	3	21.795	2.526	0.07	0.136
	Aging	19.399	1	19.399	2.249	0.140	0.045
	Restoration * Aging	48.940	3	16.313	1.891	0.144	0.106
Dentin	Restoration	130.386	3	43.462	2.032	0.122	0.113
	Aging	43.261	1	43.261	2.023	0.161	0.040
	Restoration * Aging	43.537	3	14.512	0.679	0.569	0.041

The MGW data showed a predominance of zero values across the tested groups, indicating that no detectable gap was present at many of the evaluated sites. Despite the zero median values observed in most groups, the ranges demonstrated variability, with measurable gaps detected in some specimens. This zero-heavy distribution represents an important analytical characteristic of the dataset and should be considered when interpreting rank-based comparisons, particularly given the small subgroup size (*n* = 7). The Kruskal–Wallis test revealed no statistically significant differences in MGW between immediate and aged conditions for any of the tested restorative materials (*p* > 0.05). Similarly, no statistically significant differences in MGW were detected among the four bulk-fill restorative materials at either enamel or dentin margins, either immediately or after aging (*p* > 0.05), Table 3. Additionally, Mann–Whitney U test revealed no statistically significant difference in external MGW between the tooth substrate types (enamel and dentin) (Z = 1.148, *p* = 0.251). Spearman correlation analysis revealed a significant negative correlation between external %CM and MGW values for both enamel (*r* = − 0.970, *p* < 0.05) and dentin (*r* = − 0.917, *p* < 0.05).

**Table 3:** Results of the Kruskal–Wallis test of external maximum gap width for materials and aging effect

Substrate	Aging	Material							*P* value
Enamel		Flowable	Preheated			SonicFill		Tetric	
	Immediate	0(0–7)	0(0–7)			0(0–0)		0(0–0)	1.0
	Delayed	0(0–7)	0(0–4)			0(0–0)		0(0-9.2)	0.27
	*P* value (Aging)	1.0	0.27		1.0			0.07	
	Overall comparison Kruskal–Wallis test H = 3.904, df = 3, *P* = 0.078, ε² = 0.017 among materials								
Dentin									
	Immediate	0(0–7)		0(0–0)	0(0–0)		0(0-16.3)		0.079
	Delayed	0(0-15.13)		0(0–30)	0(0–0)		0(0-62.04)		0.070
	*P* value (Aging)	1.0		0.070	1.0		0.079		
	Overall Comparison Kruskal–Wallis H = 7.057, df = 3, *P* = 0.079, ε² = 0.169 among materials								

Data expressed as median (min-max)

### Internal marginal adaptation results

The two-way ANOVA revealed no statistically significant effects of restorative material, aging, or their interaction on the percentage of continuous margins (% CM) (*p* > 0.05) Table 4. Similar to the external MGW measurements, internal MGW showed a predominance of zero values, reflecting the absence of detectable gaps at many evaluated sites, while the ranges indicated measurable gaps in some specimen. According to Table 5, the overall effect of aging on internal MGW was statistically significant (*p* < 0.05), with MGW values being significantly lower in the immediate groups than in the aged group, however individual comparisons within each material did not reach statistical significance. In addition, significant differences were observed among the tested materials at the dentin-gingival margin (*p* < 0.05). Pairwise post-hoc comparisons showed significant differences between Flowable and Tetric (unadjusted *P* = 0.002, Bonferroni-adjusted *P* = 0.012) and between SonicFill and Tetric (unadjusted *P* = 0.003, Bonferroni-adjusted *P* = 0.015). Although the comparison between Preheated and Tetric was statistically significant before adjustment (*P* = 0.019), it did not remain statistically significant after Bonferroni correction (adjusted *P* = 0.113). No significant differences were observed among the other tested material pairs (adjusted *P* > 0.05), as shown in Table 6 (pairwise comparisons).

**Table 4:** Results of Two-way ANOVA test of internal percentage of continuous margin across materials and aging

Source	Type III Sum of Squares	df	Mean Square	F	Sig.	Partial Eta Squared η²
Restoration	133.817	3	44.606	0.739	0.534	0.044
Aging	242.362	1	242.362	4.014	0.051	0.077
Restoration * Aging	37.573	3	12.524	0.207	0.891	0.013

**Table 5:** Results of the Kruskal–Wallis test of internal maximum gap width for materials and aging effect

Aging	Material				*P* value
	Flowable	Preheated	SonicFill	Tetric	
Immediate	0(0–7)	0(0–0)	0(0–0)	0(0-16.3)	0.14
Delayed	0(0-15.13)	0(0–30)	0(0–0)	0(0-62.04)	0.006*
P value (Aging)	1.0	0.07	1.0	0.079	
Overall Aging	Kruskal Wallis H = 5.829, df = 1, *P* = 0.016*, ε² = 0.089				
Overall comparison among materials	Kruskal–Wallis H = 12.587, df = 3, *P* = 0.006*, **ε² =** 0.184				

Data described as median (min-max)

*statistically significant

ε² represents epsilon-squared effect size

**Table 6:** Pairwise comparisons of internal marginal gap width between restorative materials following the Kruskal–Wallis test with Bonferroni adjustment

(I) Restoration	(J)Restoration	Test Statistic	Std. Error	Z score	*P* value	Adjusted *P* (Bonferroni)	Effect size (*r*)
flowable bulk -fill composite	preheated	-3.357	4.561	-0.736	0.462	1.000	0.098
	Sonic	-0.286	4.561	-0.063	0.950	1.000	0.008
	Tetric	-14.071	4.561	-3.085	0.002*	0.012*	0.412
preheated bulk-fill composite	Sonic	3.071	4.561	0.673	0.501	1.000	0.090
	Tetric	-10.714	4.561	-2.349	0.019*	0.113	0.314
Sonic bulk-fill composite	Tetric	-13.786	4.561	-3.023	0.003*	0.015*	0.404

Effect size was calculated as $\mathrm{r}=\mid\mathrm{Z}\mid/\mathrm{Nr}=\mid\mathrm{Z}\mid/\sqrt{N}$

*P*-values were adjusted using Bonferroni correction

*Statistically significant

**Fig. 1:**
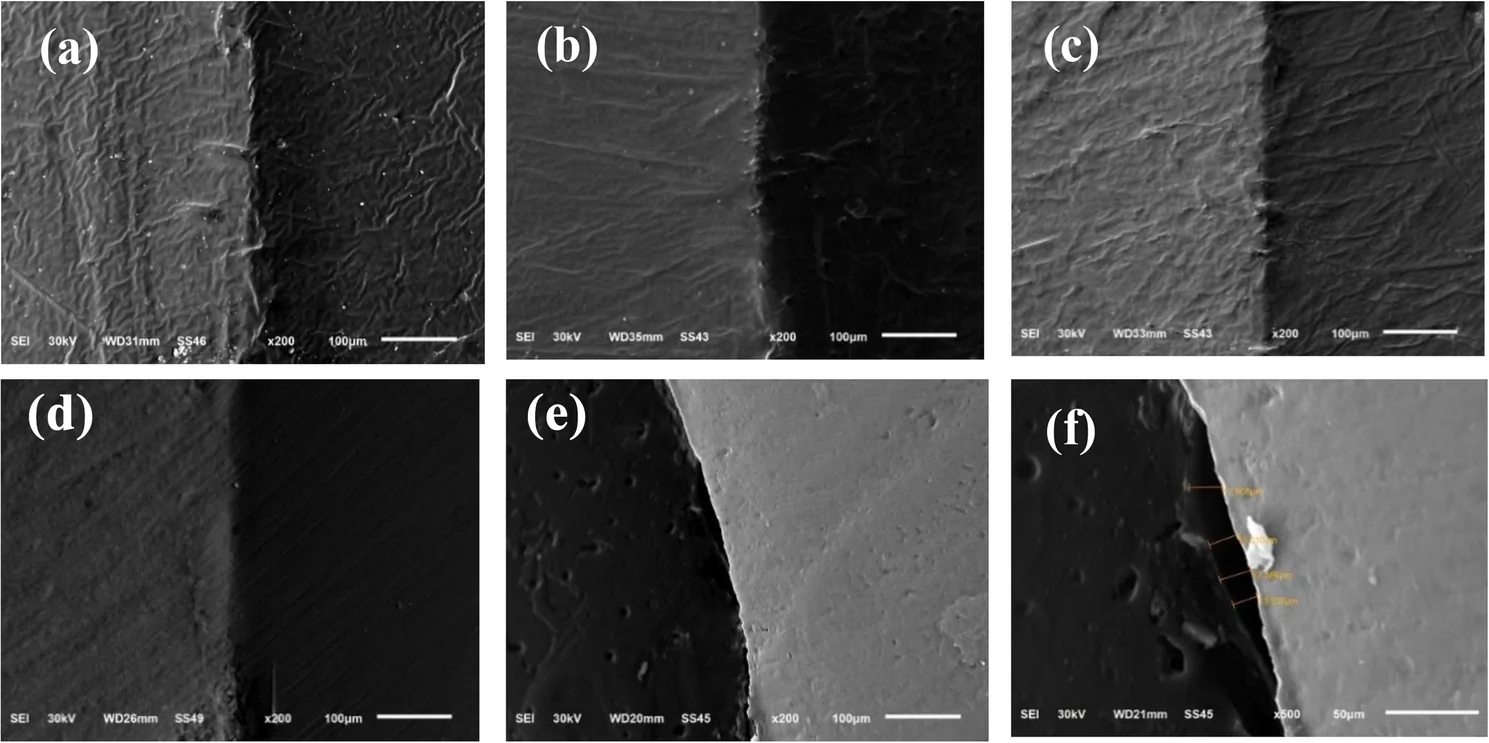
Representative SEM micrographs. of external adaptation evaluation of the aged four tested bulk-fill restorative materials. **a**: Bulk Flow composite// proximal enamel interfaces at 200×. **b**: Preheated Viscalor composite // proximal enamel interfaces at 200×. **c**: SonicFill composite// proximal enamel interfaces at 200×. **d**–**f**: Tetric Bulk-fill composite// proximal enamel interfaces at 200×,200x and 500× respectively

**Fig. 2:**
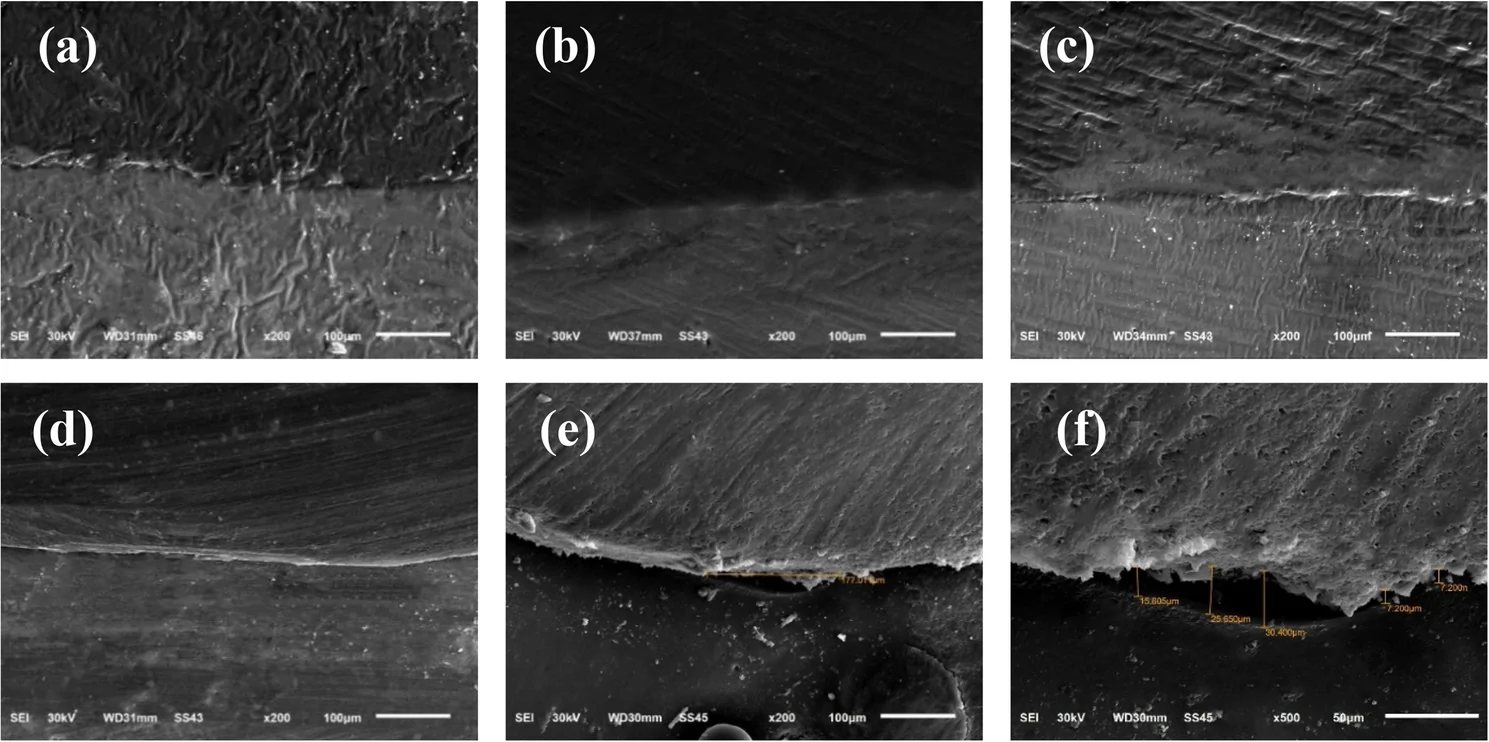
Representative SEM micrographs: **a**: Bulk Flow composite// gingival dentin interfaces at 200×. **b**: Preheated Viscalor composite // gingival dentin interfaces at 200×. **c**: SonicFill composite// gingival dentin interfaces at 200×. **d**–**f**: Tetric Bulk-fill composite// gingival dentin interfaces at 200×, 200x and 500× respectively

**Fig. 3:**
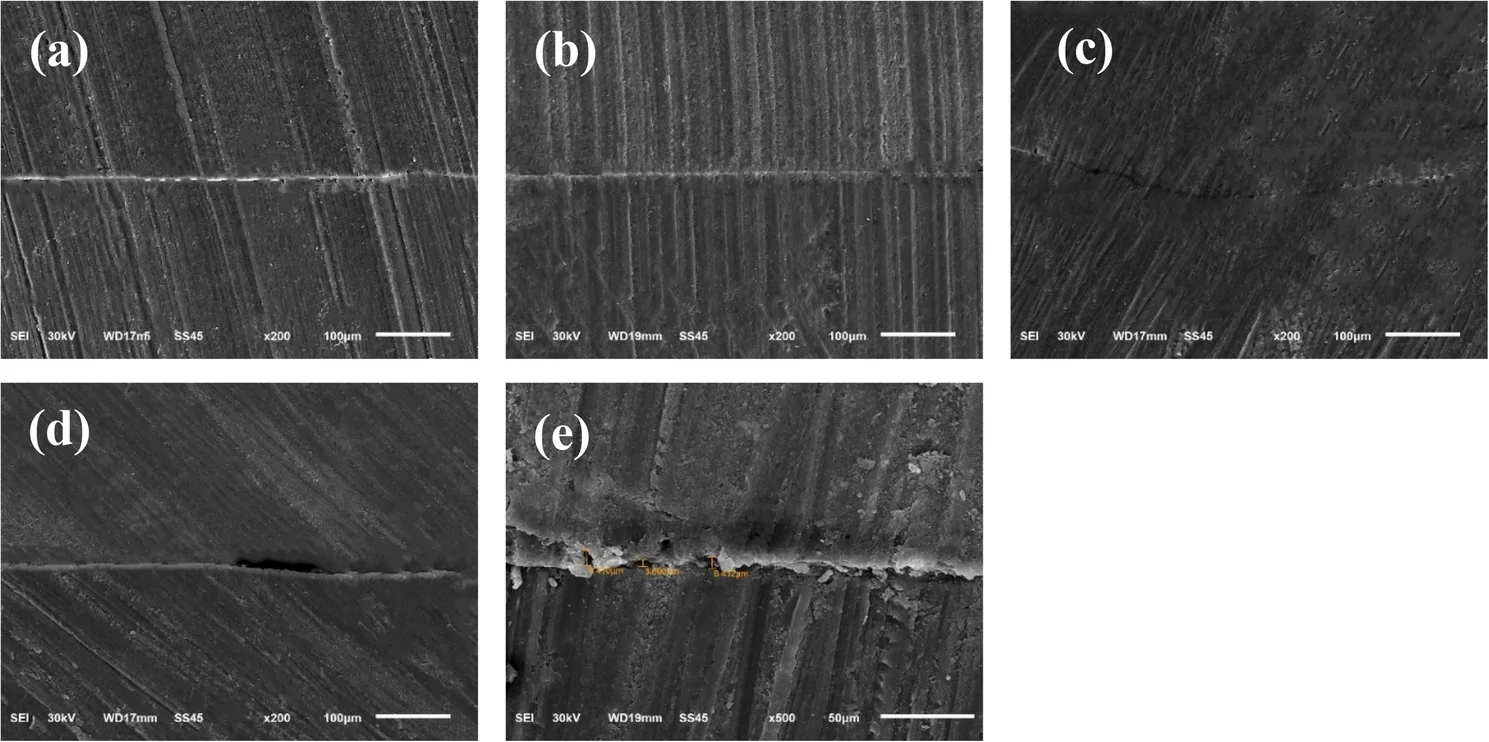
Representative SEM micrographs of internal adaptation evaluation of the aged four tested bulk-fill restorative materials. **a**: Bulk Flow composite// gingival interfaces at 200×. **b**: Preheated Viscalor composite // gingival interfaces at 200×. **c**: SonicFill composite// gingival at 200×. **d** and **e**: Tetric Bulk-fill composite// gingival interfaces at 200×and 500× respectively

**Fig. 4:**
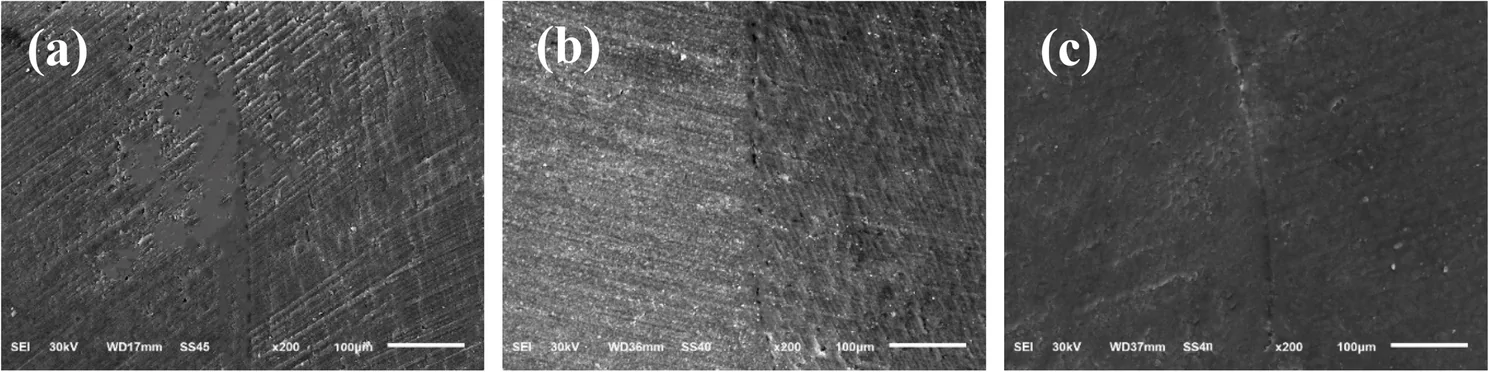
Representative SEM micrographs of internal adaptation evaluation of the four tested bulk-fill restorative materials. **a**: Bulk Flow composite// axial interfaces at 200×. **b**: Preheated Viscalor composite // axial interfaces at 200×. **c**: SonicFill composite// axial interfaces at 200×

Spearman correlation analysis demonstrated a strong negative correlation between internal %CM and MGW values (*r* = − 0.992, *p* < 0.05). Furthermore, significant positive correlations were observed between external and internal %CM values (*r* = 0.587, *p* < 0.05) and between external and internal MGW values (*r* = 0.598, *p* < 0.05). Representative SEM images for external and internal adaptation evaluations with different magnifications are presented in Figs. 1, 2, 3 and 4.

## Discussion

Adaptation is a critical factor that contributes to the durability of any restoration within the oral cavity. Both external and internal adaptation are affected by several parameters, including material viscosity, composition, insertion technique, and polymerization shrinkage stresses. Bulk-fill resin composites were introduced to simplify restorative procedures and reduce polymerization shrinkage stresses [6]. In addition, they eliminate the need for incremental layering required in conventional composites [11]. Various approaches, such as flowable formulations, preheating, and sonic activation, have been proposed to improve handling characteristics and adaptation to cavity walls [20, 32]. In the present study, four bulk-fill resin composites with different viscosity characteristics were evaluated for their marginal and internal adaptation immediately and after artificial aging using SEM analysis.

The adaptation of any restorative material to the cavity margins can be measured using either microleakage and dye penetration tests or marginal adaptation measurements. In this investigation, a quantitative method was adopted to assess marginal continuity, gap presence, and gap width, providing an objective assessment compared with more conventional qualitative approaches. The use of epoxy resin replicas allowed standardized evaluation and enabled correlation between external and internal adaptation, although minor distortions associated with the replication process cannot be completely excluded [31]. 

Standardization of the experimental conditions was attempted by selecting molars of comparable size and preparing standardized Class II cavities with similar dimensions. This was intended to minimize variability and approximate a consistent C-factor among specimens. Artificial aging was performed using water storage (12 months) combined with thermocycling. The justification for selecting both approaches was to simulate hydrolytic and thermal stresses resulting from mismatches in the coefficients of thermal expansion (CTE) between the tooth and the restorative material associated with long-term oral exposure [31]. However, mechanical fatigue loading was not included, which limits the ability of this protocol to fully reproduce the clinical oral environment. Future studies incorporating mechanical loading are warranted.

In the current study, aging had no impact on external marginal integrity (% CM and MGW), while it significantly affected internal marginal integrity (MGW); thus, the first null hypothesis has not been rejected with respect to external adaptation but was rejected for internal MGW. The type of the tested resin composite and tooth substrate (enamel vs. dentin) had no impact on % CM and MGW values of external adaptation, therefore, the second null hypothesis has not been rejected. According to internal adaptation, composite type did not significantly affect % CM values, while the type of bulk-fill resin composite material affected the MGW results; therefore, the third null hypothesis has not been rejected for % CM. Conversely, it was rejected for internal marginal integrity (MGW). Furthermore, a significant correlation was observed between external marginal and internal adaptation parameters (% CM and MGW), leading to the rejection of the fourth null hypothesis.

The results of the present study indicated that the applied aging protocol did not significantly affect external marginal adaptation, while a parameter-specific effect was observed for internal adaptation, where internal MGW values were significantly increased after aging. This finding suggests that internal adaptation may be more susceptible to the effects of thermocycling and water storage compared with marginal adaptation. The absence of significant changes in marginal adaptation may be attributed to the stability of the adhesive interface and the effectiveness of the bonding protocol. In contrast, the increase in internal MGW values following aging could be related to hydrolytic degradation at the tooth-restoration interface or stress development within the bulk of the material over time [33]. 

A possible explanation is that internal MGW may be more sensitive to subtle changes occurring at the internal tooth-restoration interface during prolonged water storage and thermocycling. The internal dentin-gingival region is exposed to a more challenging bonding environment and may be more susceptible to stress accumulation resulting from hydrolytic degradation, water sorption, polymerization-related contraction, and repeated thermal expansion and contraction. These effects may increase the width of preexisting microscopic interfacial discrepancies without necessarily producing a measurable reduction in the overall percentage of continuous margins. The interpretation of the MGW findings should nevertheless be made cautiously because of the predominance of zero values, which represents an important analytical characteristic of the dataset, and the small subgroup size (*n* = 7), which may limit the sensitivity and granularity of rank-based comparisons to detect subtle differences between groups. In contrast, % CM represents the proportion of the interface that remains continuously adapted and may be less sensitive to small increases in the width of localized gaps. The significant increase in internal MGW without a corresponding significant change in internal % CM may therefore indicate that aging affected the magnitude of localized gaps rather than the overall continuity of the interface.

Among the tested materials, PALFIQUE^®^ Bulk Flow, SonicFill™ 3, and VisCalor Bulk demonstrated numerically different adaptation values compared with those of the high-viscosity bulk-fill composite (Tetric N-Ceram Bulk Fill). However, most between-material differences did not reach statistical significance. Therefore, these numerical differences should be interpreted as trends rather than evidence of superior adaptation or clinical performance. This finding may be partly related to the flow characteristics of the tested materials, as lower viscosity and viscosity-modification strategies such as sonic activation and preheating may facilitate adaptation to cavity walls. However, these characteristics were not directly evaluated in the present study [34, 35]. 

PALFIQUE^®^ Bulk Flow is a No-cap, highly filled flowable composite characterized by relatively low polymerization shrinkage stress and a low modulus of elasticity, properties that may contribute to its flow and adaptation to irregular cavity margins. For SonicFill™ 3, sonic activation can substantially reduce the viscosity of the composite, thereby increasing its flowability and facilitating adaptation to the cavity walls [25, 26]. Similarly, preheating of VisCalor Bulk decreases its viscosity and increases its flow capacity, which may facilitate adaptation during placement [20, 22]. 

The high-viscosity bulk-fill composite exhibited comparatively lower adaptation values, which may be related to its handling characteristics and resistance to flow during placement. Nevertheless, given that several comparisons did not reach statistical significance, these differences should be interpreted as trends rather than definitive differences in performance. These findings are in agreement with previous studies [15, 17] reporting that modifications in viscosity and handling characteristics, including preheating and sonic activation, may influence marginal adaptation. Additionally, comparable adaptation was observed between enamel and dentin substrates, suggesting that the tested materials may perform similarly under different cavity substrate conditions.

For the internal adaptation analysis, variations in maximum gap width were observed among the tested materials, whereas % CM values remained generally comparable. These differences in gap measurements may reflect variations in material adaptation behavior during polymerization [28]. According to the literature, volumetric shrinkage of bulk-fill resin composites generally ranges between approximately 1–3%, with lower values reported for SonicFill™ and thermo-viscous or preheated composites such as VisCalor. In contrast, conventional high-viscosity bulk-fill composites such as Tetric N-Ceram Bulk Fill have been reported to exhibit higher shrinkage values, reaching approximately 4% in previous study [6]. Lower polymerization shrinkage and shrinkage stress may reduce contraction-related stress development at the bonded tooth–restoration interface, potentially contributing to more favorable internal adaptation.

In addition to polymerization-related effects, the enhanced flow of lower-viscosity materials may facilitate more intimate adaptation to the internal cavity walls and reduce the formation of interfacial voids during placement. A relatively lower elastic modulus may further provide greater elastic compliance, allowing limited deformation and redistribution of stresses generated by repeated thermal expansion and contraction during thermocycling. Consequently, lower stress concentration at the bonded interface may reduce the potential for interfacial separation and subsequent internal gap formation during aging. Thus, the combined effects of improved initial adaptation, lower polymerization shrinkage stress, and greater elastic compliance may have contributed to the more favorable internal adaptation observed for some of the tested materials. However, these mechanisms should be considered possible explanations rather than direct findings, as flow characteristics, elastic modulus, polymerization shrinkage stress, and interfacial debonding were not directly measured in the present study.

Importantly, the observed differences in internal adaptation should not be attributed solely to material viscosity. The tested bulk-fill composites differ in several material-specific characteristics beyond viscosity, including filler composition and loading, resin matrix formulation, polymerization chemistry, and optical properties. These factors may independently influence polymerization behavior, shrinkage stress development, flow, and stress distribution within the restoration. Therefore, the more favorable internal adaptation observed for some of the lower-viscosity materials may reflect the combined effect of their overall material formulations rather than viscosity alone. Since the present study compared commercially available materials with multiple compositional differences, the independent effect of viscosity could not be isolated.

The strong negative correlations observed between %CM and MGW are expected, as these parameters assess complementary aspects of marginal adaptation: a higher % CM indicates a greater proportion of continuous margins, whereas a higher MGW indicates a greater marginal gap. As both parameters were obtained from the same specimens and reflect related characteristics of marginal adaptation, their correlation represents an association between complementary measures rather than independent biological validation. Similarly, the correlations observed between external and internal adaptation measures may reflect the interrelationship between these measurements within the same specimens. Therefore, these correlations should be interpreted as measures of association and should not be considered evidence of causation or as independent biological validation of either adaptation measurement.

This study has some limitations that should be acknowledged. Although the sample size was determined by an a priori power analysis, the relatively small subgroup sample size (*n* = 7), may have increased the risk of Type II error and may limit detection of subtle intergroup differences. The aging protocol was limited to water storage and thermocycling without the inclusion of mechanical loading, which may not fully replicate intraoral functional conditions. Future studies incorporating mechanical fatigue loading in combination with thermocycling and water storage may provide a more comprehensive assessment of long-term restoration performance. Additionally, although SEM analysis was performed using standardized procedures, measurement bias cannot be completely excluded due to the technique-sensitive nature of image analysis. The use of epoxy resin replicas, although widely accepted, may introduce minor distortions during impression and replication procedures. Another limitation of the present study is that each restorative material was used with its corresponding manufacturer-recommended adhesive system. Differences in adhesive composition and chemical characteristics may have contributed to the observed differences in marginal adaptation and may therefore represent a potential confounding factor. Consequently, the effects observed in the present study cannot be attributed exclusively to differences in composite viscosity. Future investigations using a standardized adhesive system across the tested restorative materials may help isolate the specific contribution of composite viscosity and material-related properties. Finally, as an in vitro study, the findings may not be directly extrapolated to clinical conditions, and caution should be exercised when interpreting the result.

## Clinical relevance

This in vitro study found generally comparable external marginal adaptation among the tested bulk-fill resin composites under the simulated aging conditions. However, differences among materials were observed for internal MGW, indicating that some material-specific differences may occur at the internal tooth-restoration interface. Further studies incorporating mechanical fatigue and clinical evaluation are warranted.

## Conclusions

Within the limitations of this study, the following conclusions can be drawn:

1. The applied aging protocol did not significantly affect external marginal adaptation, whereas internal MGW increased significantly after aging.
2. Lower-viscosity composite restorative systems showed numerically lower gap values, but most differences were not statistically significant. A statistically significant difference among materials was observed only for internal MGW.
3. External marginal and internal adaptation measurements were significantly correlated.

## Supplementary Information

Supplementary Material 1.

## Data Availability

The data that support the findings of this study are available from the corresponding author upon reasonable request.

## Abbreviations

SEM: Scanning electron microscopy
% CM: Percentage of continuous margin
MGW: Maximum gap width
