# Supplementary material for: Effect of aging on marginal and internal adaptation of bulk-fill resin composites with different viscosities in class II restorations
Source: BMC Oral Health. 2026 Nov 5;26:2193. doi: 10.1186/s12903-026-10102-1 (PMC13737557; doi:10.1186/s12903-026-10102-1)
Supplement: Supplementary file 1 [file 12903_2026_10102_MOESM1_ESM.docx]

**Supplementary Table S1:** Results of the person Chi-square analysis of external percentage of continues margin across materials and aging.

| **Substrate** | **Aging / Material** | **81% CM** | **85.78% CM** | **85.55% CM** | **85.87% CM** | **87.10% CM** | **90% CM** | **97.90% CM** | **100% CM** | **Statistical Test** | **X^2^** | **P-value** |
| --- | --- | --- | --- | --- | --- | --- | --- | --- | --- | --- | --- | --- |
|  |  |  |  |  |  |  |  |  |  |  |  |  |
| Enamel | Immediate | 0  (0.0%) | 0  (0.0%) | 0  (0.0%) | 1  (0.5%) | 0  (0.0%) | 0  (0.0%) | 0  (0.0%) | 27  (26.5%) | Chi-square | 3.170 | 0.366 |
|  | Delayed | 1  (0.5%) | 1  (0.5%) | 1  (0.5%) | 0  (0.0%) | 0  (0.0%) | 0  (0.0%) | 0  (0.0%) | 25  (26.6%) |  |  |  |
|  | Flowable Bulk-fill | 0  (0.0%) | 0  (0.0%) | 0  (0.0%) | 0  (0.0%) | 0  (0.0%) | 0  (0.0%) | 0  (0.0%) | 14  (13.3%) | Chi-square | 9.208 | 0.418 |
|  | Preheated Bulk-fill | 0  (0.0%) | 0  (0.0%) | 0  (0.0%) | 1  (0.3%) | 0  (0.0%) | 0  (0.0%) | 1  (0.3%) | 13  (12.8%) |  |  |  |
|  | Sonic Bulk-fill | 0  (0.0%) | 0  (0.0%) | 0  (0.0%) | 0  (0.0%) | 0  (0.0%) | 0  (0.0%) | 0  (0.0%) | 14  (13.3%) |  |  |  |
|  | Tetric Bulk-fill | 1  (0.3%) | 1  (0.3%) | 0  (0.0%) | 0  (0.0%) | 0  (0.0%) | 0  (0.0%) | 0  (0.0%) | 12  (12.8%) |  |  |  |
| Dentin | Immediate | 0  (0.0%) | 0  (0.0%) | 0  (0.0%) | 1  (0.5%) | 0  (0.0%) | 0  (0.0%) | 1  (0.5%) | 26  (25.5%) | Chi-square | 5.176 | 0.395 |
|  | Delayed | 1  (0.5%) | 1 (0.5%) | 1  (0.5%) | 0  (0.0%) | 1  (0.5%) | 1  (0.5%) | 0  (0.0%) | 24  (25.5%) |  |  |  |
|  | Flowable Bulk-fill | 0  (0.0%) | 0  (0.0%) | 0  (0.0%) | 0  (0.0%) | 0  (0.0%) | 0  (0.0%) | 0  (0.0%) | 14  (13.3%) | Chi-square | 15.843 | 0.393 |
|  | Preheated Bulk-fill | 1  (0.3%) | 0  (0.0%) | 0  (0.0%) | 0  (0.0%) | 0  (0.0%) | 0  (0.0%) | 1  (0.3%) | 13  (12.8%) |  |  |  |
|  | Sonic Bulk-fill | 0  (0.0%) | 0  (0.0%) | 0  (0.0%) | 0  (0.0%) | 0  (0.0%) | 0  (0.0%) | 0  (0.0%) | 14  (13.3%) |  |  |  |
|  | Tetric Bulk-fill | 0  (0.0%) | 0  (0.0%) | 1  (0.3%) | 1  (0.3%) | 1  (0.3%) | 1  (%) | 0  (0.0%) | 10  (12.8%) |  |  |  |
|  |  |  |  |  |  |  |  |  |  |  |  |  |

**Supplementary Table S2:** Results of T-test representing the effect of substrate on external %CM values

| **Independent Samples Test** | | | | | | | | | | |  |
| --- | --- | --- | --- | --- | --- | --- | --- | --- | --- | --- | --- |
|  | | **Levene's Test for Equality of Variances** | | **t-test for Equality of Means** | | | | | | |  |
|  |  | F | Sig. | t | Df | Sig. (2-tailed) | Mean Difference | Std. Error Difference | 95% Confidence Interval of the Difference | | |
|  |  |  |  |  |  |  |  |  | Lower | Upper | |
| **Enamel.CM %** | Equal variances assumed | 4.002 | 0.05 | .988 | 110 | 0.33 | 0.75 | 0.76 | 0.76 | 2.26 | |
|  | Equal variances not assumed |  |  | .988 | 95.456 | 0.32 | 0.75 | 0.76 | 0.76 | 2.26 | |

**Supplementary Table S3:** Mann–Whitney U test comparing internal marginal gap width (MGW) between tooth substrates

|  |  | **Mean** | **Std. Deviation** | **Minimum** | **Maximum** | **Percentiles** | | |  |
| --- | --- | --- | --- | --- | --- | --- | --- | --- | --- |
|  |  |  |  |  |  | **25th** | **50th (Median)** | **75th** |  |
| Enamel. MGW |  | 2.27 | 9.92 | 0.00 | 62.04 | .0000 | .0000 | .0000 |  |
| Dentin. MGW |  | 4.16 | 13.73 | 0.00 | 62.04 | .0000 | .0000 | .0000 |  |
| Z | 1.148 | | | | | | | | |
| P Value | 0.251 | | | | | | | | |

Data are expressed as Mean ± SD, Minimum–Maximum, and Percentiles.

**Supplementary Table S4:** Results of the person Chi-square analysis of internal percentage of continues margin across materials and aging.

| **Aging**  **/Materials** | **52%** | **71%** | **73%** | **75%** | **80%** | **84%** | **85%** | **89.1%** | **94%** | **96%** | **100%** | **Statistical Test** | **X^2^** | **P-value** |
| --- | --- | --- | --- | --- | --- | --- | --- | --- | --- | --- | --- | --- | --- | --- |
| Immediate (I) | 0  (0.0%) | 0  (0.0%) | 0  (0.0%) | 0  (0.0%) | 0  (0.0%) | 0  (0.0%) | 0  (0.0%) | 0  (0.0%) | 2  (2.4%) | 1  (0.5%) | 25 | Chi-square test | 14.14 | 0.16 |
| Delayed (D) | 1  (1.2%) | 1  (1.2%) | 1  (1.2%) | 1  (1.2%) | 2  (2.4%) | 1  (1.2%) | 2  (2.4%) | 1  (1.2%) | 0  (0.0%) | 0  (0.0%) | 18 |  |  |  |
| Flowable | 0  (0.0%) | 0  (0.0%) | 0  (0.0%) | 1  (1.2%) | 0  (0.0%) | 0  (0.0%) | 0  (0.0%) | 0  (0.0%) | 0  (0.0%) | 0  (0.0%) | 13 | Chi-square test | 38.36 | 0.54 |
| Preheated | 0  (0.0%) | 0  (0.0%) | 0  (0.0%) | 0  (0.0%) | 0  (0.0%) | 0  (0.0%) | 1  (1.2%) | 1  (1.2%) | 0  (0.0%) | 1  (1.2%) | 11 |  |  |  |
| Sonic | 00  (0.0%) | 0  (0.0%) | 0  (0.0%) | 1  (1.2%) | 0  (0.0%) | 0  (0.0%) | 0  (0.0%) | 0  (0.0%) | 0  (0.0%) | 0  (0.0%) | 13 |  |  |  |
| Tetric | 1  (1.2%) | 1  (1.2%) | 0  (0.0%) | 0  (0.0%) | 2  (3.6%) | 1  (1.2%) | 1  (1.2%) | 0  (0.0%) | 2  (3.6%) | 0  (0.0%) | 6 |  |  |  |
